# Supplementary material for: Genetic screens in isogenic mammalian cell lines without single cell cloning
Source: Nat Commun. 2020 Feb 6;11:752. doi: 10.1038/s41467-020-14620-6 (PMC7005275; doi:10.1038/s41467-020-14620-6)
Supplement: Supplementary file 1 — Supplementary Information [file 41467_2020_14620_MOESM1_ESM.pdf]

Genetic screens in isogenic mammalian cell lines without single cell cloning

DeWeirdt et al.

Supplementary Figures 1 – 17

Supplementary Tables 1 – 2

Supplementary Note 1

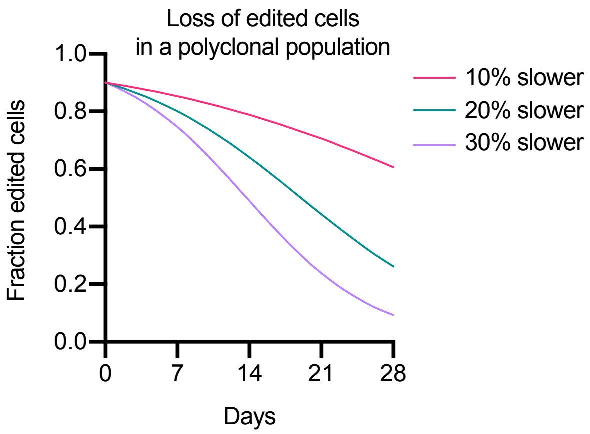

Supplementary Figure 1 Simulation of competition between edited and unedited cells in a polyclonal population. Edited cells are outcompeted over the span of 28 days assuming the edit causes cells to double 10%, 20%, or 30% more slowly than unmodified cells, and assuming the unedited cells have a doubling time of 24 hours.

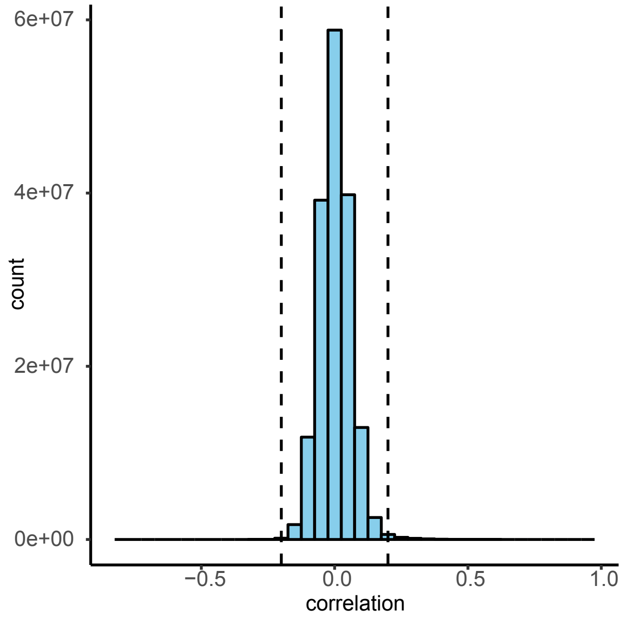

Supplementary Figure 2 Co-essentiality correlations less than -0.2 or greater than 0.2 are rare in DepMap. Histogram of all correlations using a binwidth of 0.05.

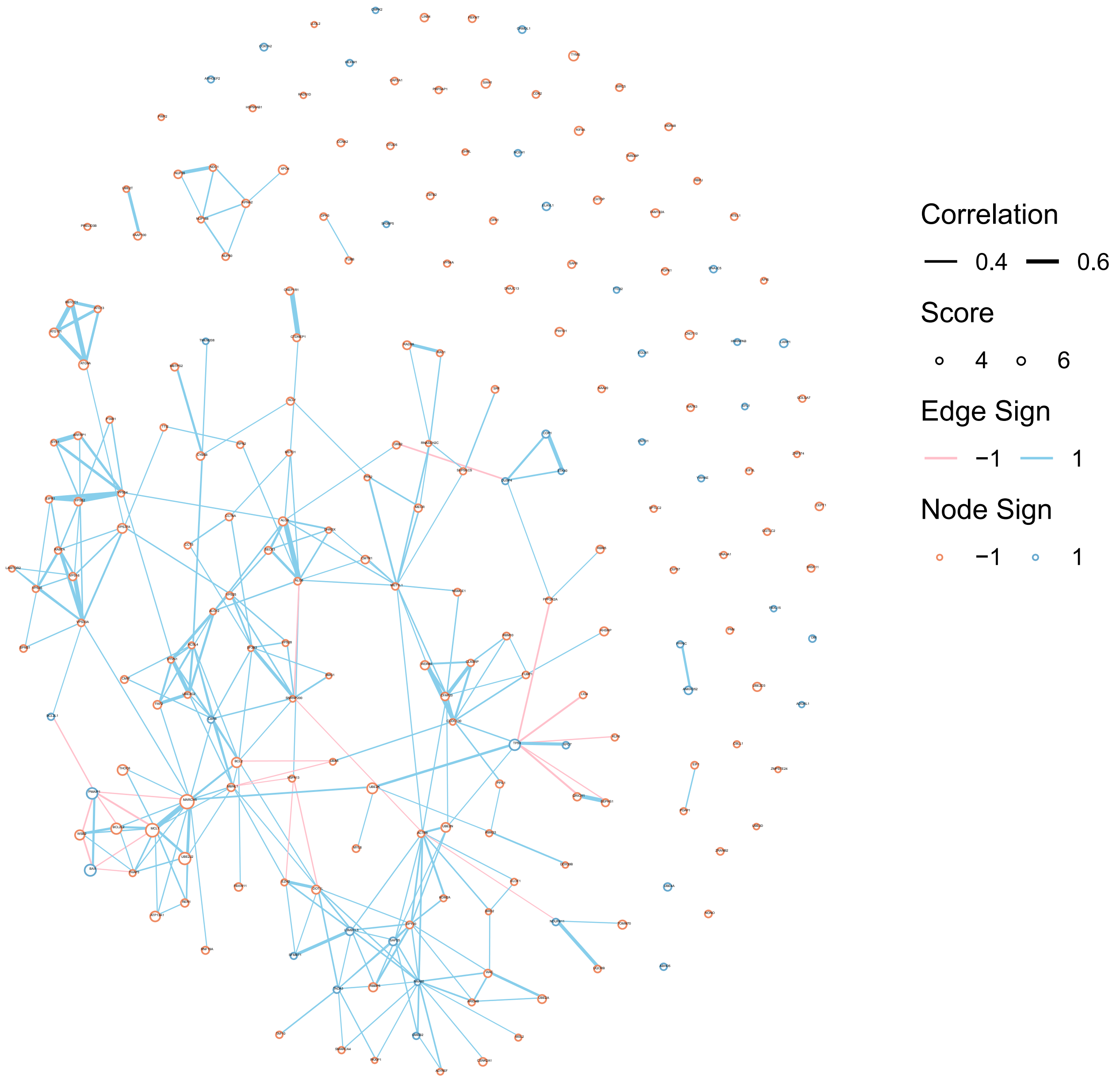

Supplementary Figure 3 Co-essentiality correlations from DepMap connect functionally related gene hits from BCL2L1 anchor screens. Nodes represent genes and the size of each node is proportional to its average Z-score across all screens. Genes with an absolute average Z-score greater than 2 across BCL2L1 conditions are included in the network. Edges represent Pearson correlations across co-essentiality profiles in DepMap. Edges are drawn between genes with an absolute correlation greater than 0.2.

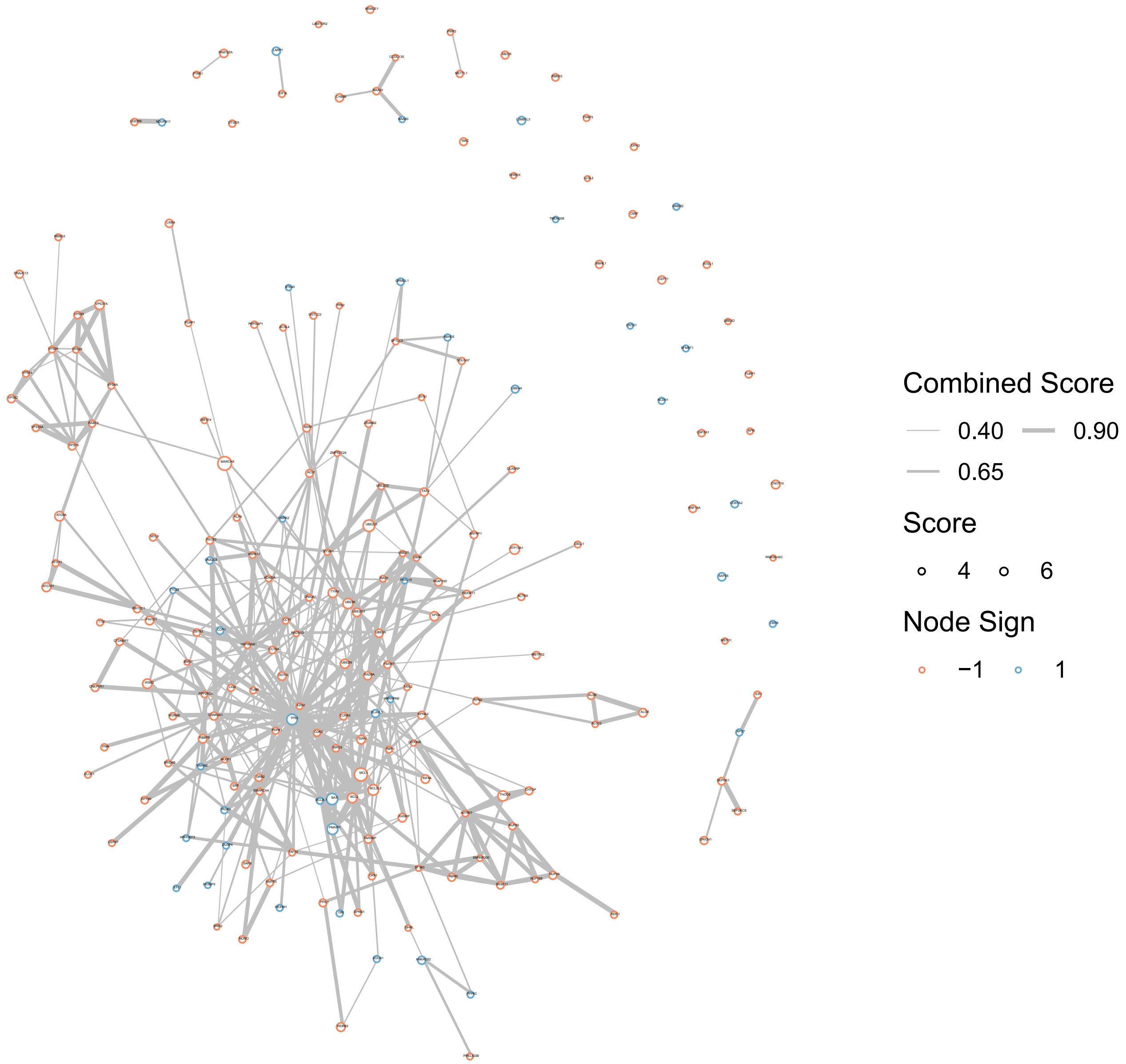

Supplementary Figure 4 Combined scores from STRING connect functionally related gene hits from BCL2L1 anchor screens. Nodes represent genes and the size of each node is proportional to its average Z-score across all screens. Genes with an absolute average Z-score greater than 2 across BCL2L1 conditions are included in the network. Edges represent combined score in STRING. Edges are drawn between genes with a STRING combined score greater than 0.4.

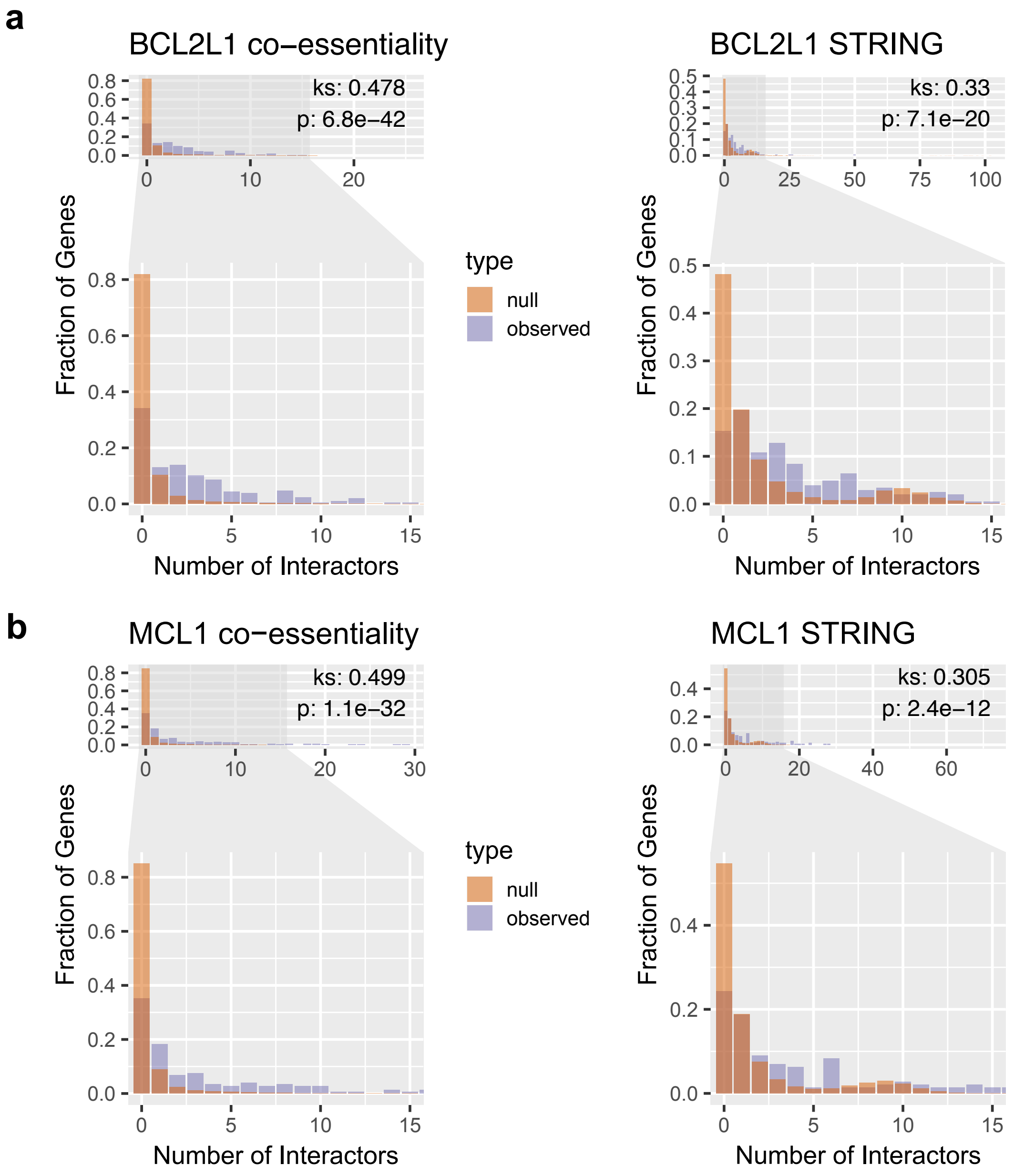

Supplementary Figure 5 Hits from the BCL2L1 and MCL1 anchor screens are enriched in interactions. (**a**) Degree distribution for the observed network of BCL2L1 hits compared with a null distribution using DepMap co-essentialities and STRING combined scores. We average 1,000 random networks, each of which has the same number of genes as the original network (n=210), to generate the null. To determine statistical significance, we used a one-sided KS test with the alternative hypothesis that the observed cumulative distribution was less than the null. (**b**) Same as (a) but for MCL1 (n=153).

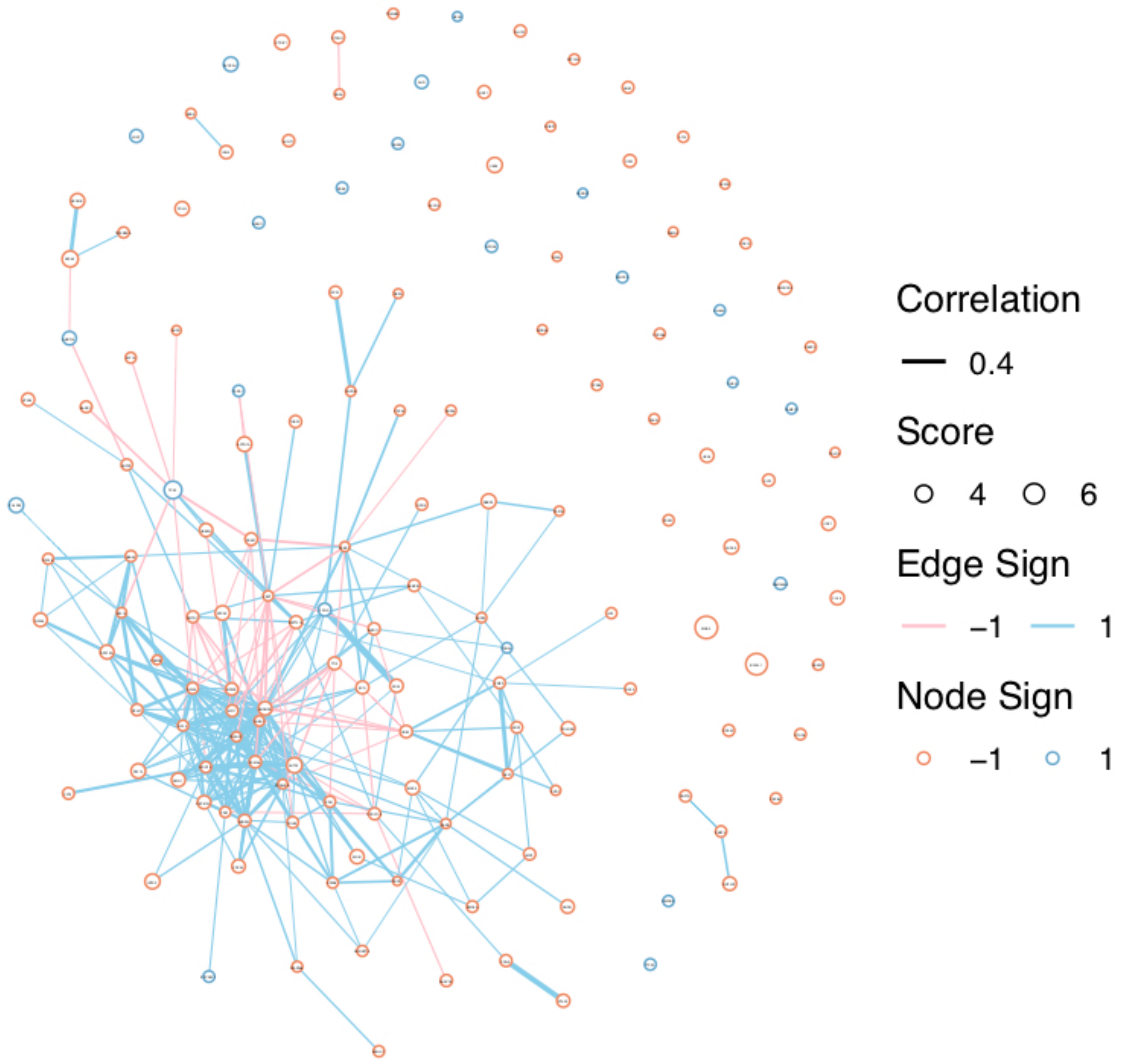

Supplementary Figure 6 Co-essentiality correlations from DepMap connect functionally related gene hits from MCL1 anchor screens. Nodes represent genes and the size of each node is proportional to its average Z-score across all screens. Genes with an absolute average Z-score greater than 2 across MCL1 conditions are included in the network. Edges represent Pearson correlations across co-essentiality profiles in DepMap. Edges are drawn between genes with an absolute correlation greater than 0.2.

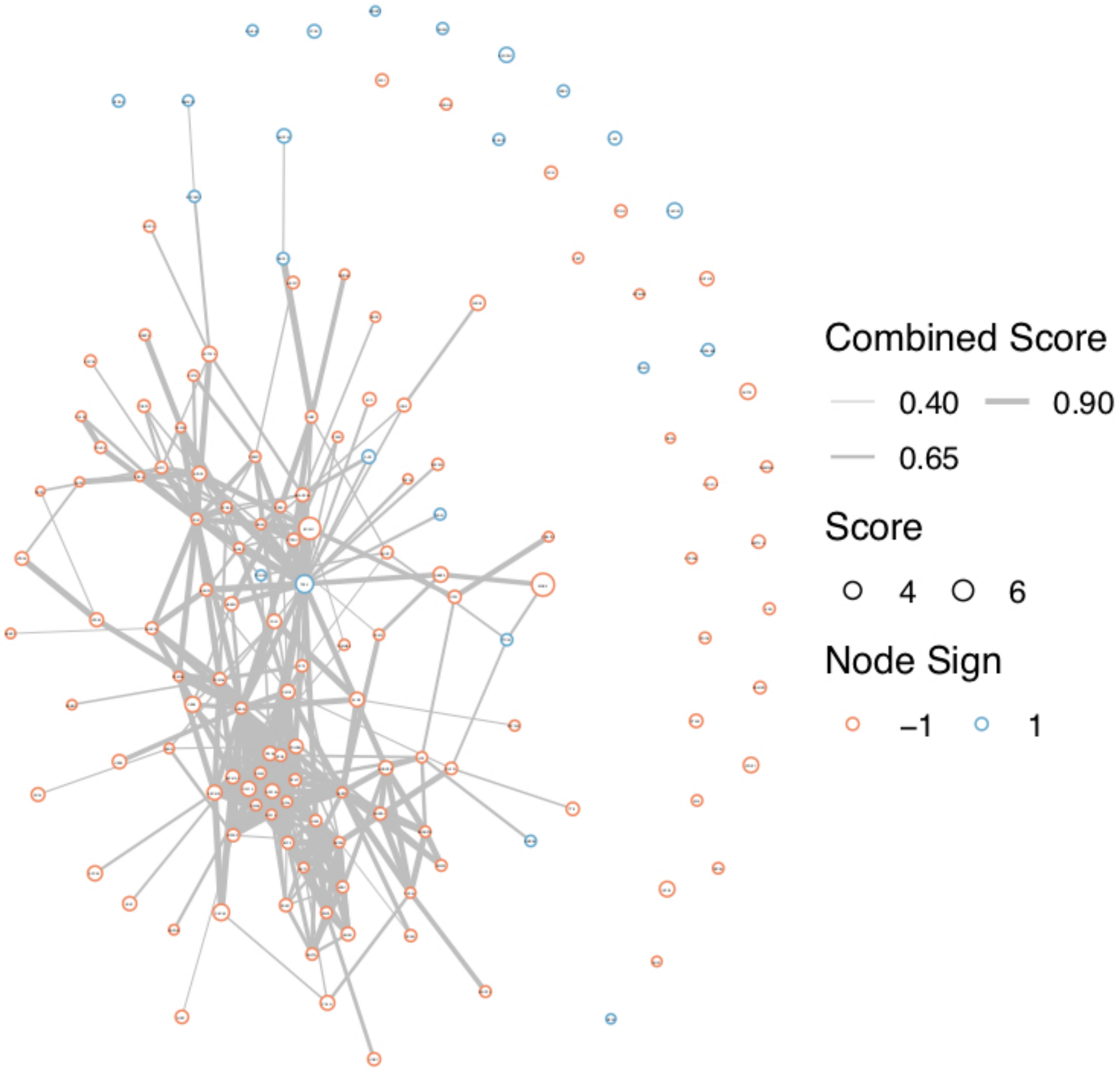

Supplementary Figure 7 Combined scores from STRING connect functionally related gene hits from MCL1 anchor screens. Nodes represent genes and the size of each node is proportional to its average Z-score across all screens. Genes with an absolute average Z-score greater than 2 across MCL1 conditions are included in the network. Edges represent combined score in STRING. Edges are drawn between genes with a STRING combined score greater than 0.4.

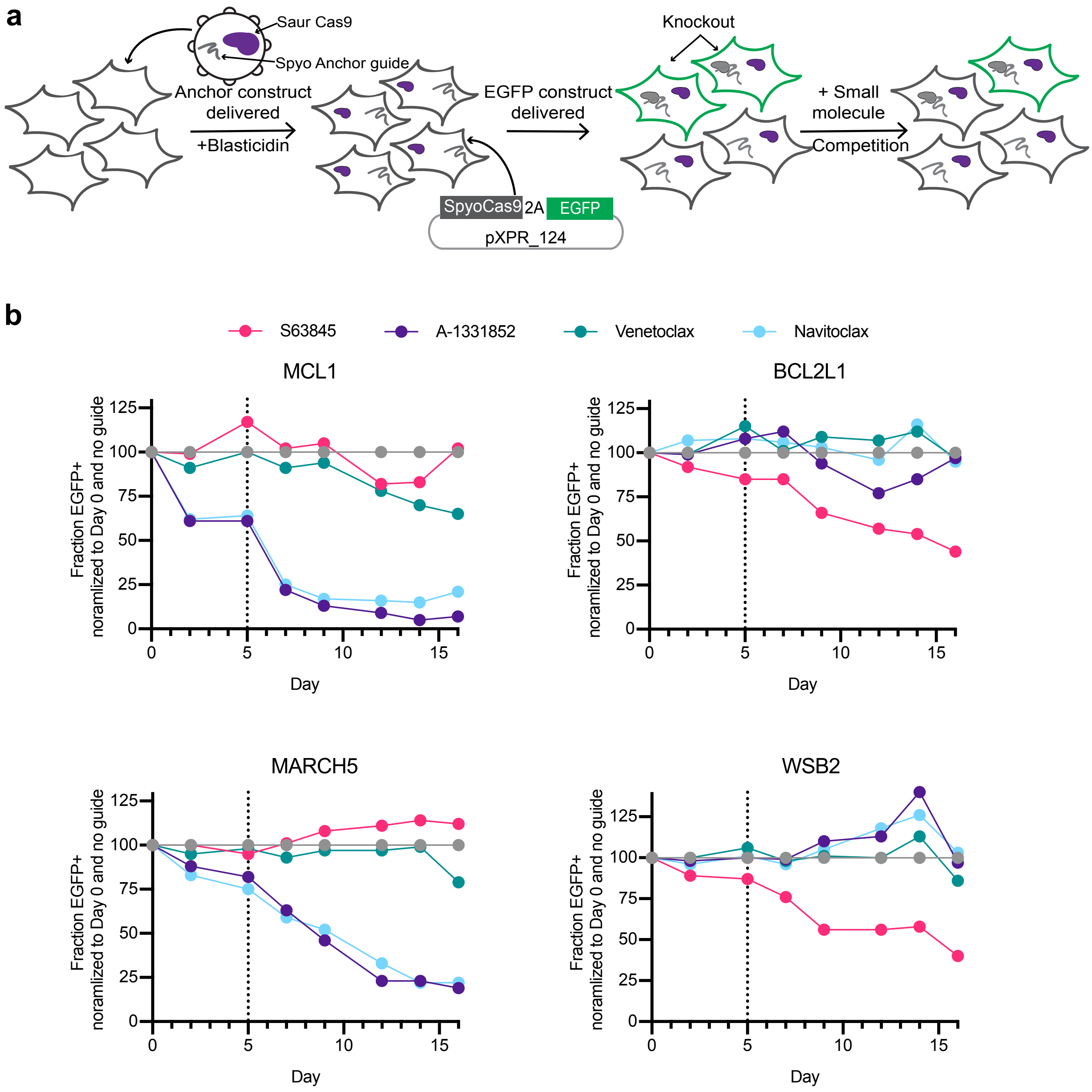

Supplementary Figure 8 EGFP competition assay validates selected genetic interactions. (**a**) Schematic of the competition experiment. First, individual Spyo-guides targeting MCL1, WSB2, BCL21, MARCH, or a control guide are delivered in a dox-inducible vector, pRDA_103. Next, Spyo-Cas9 and EGFP are co-delivered via the pXPR_124 vector, and cells are treated with small molecule inhibitors. Although SaurCas9 is delivered with the anchor vector, it is not used in this experimental set-up. (**b**) Percentage of EGFP-positive cells over time in knockout populations treated with small molecule inhibitors, normalized first to the zero time point for each guide, the time of small molecule addition, and then to the same treatment in the cells transduced with a control guide. Doxycycline was added on day 5, indicated by the vertical dotted line. n= 1.5e6 cells per population examined in a single experiment.

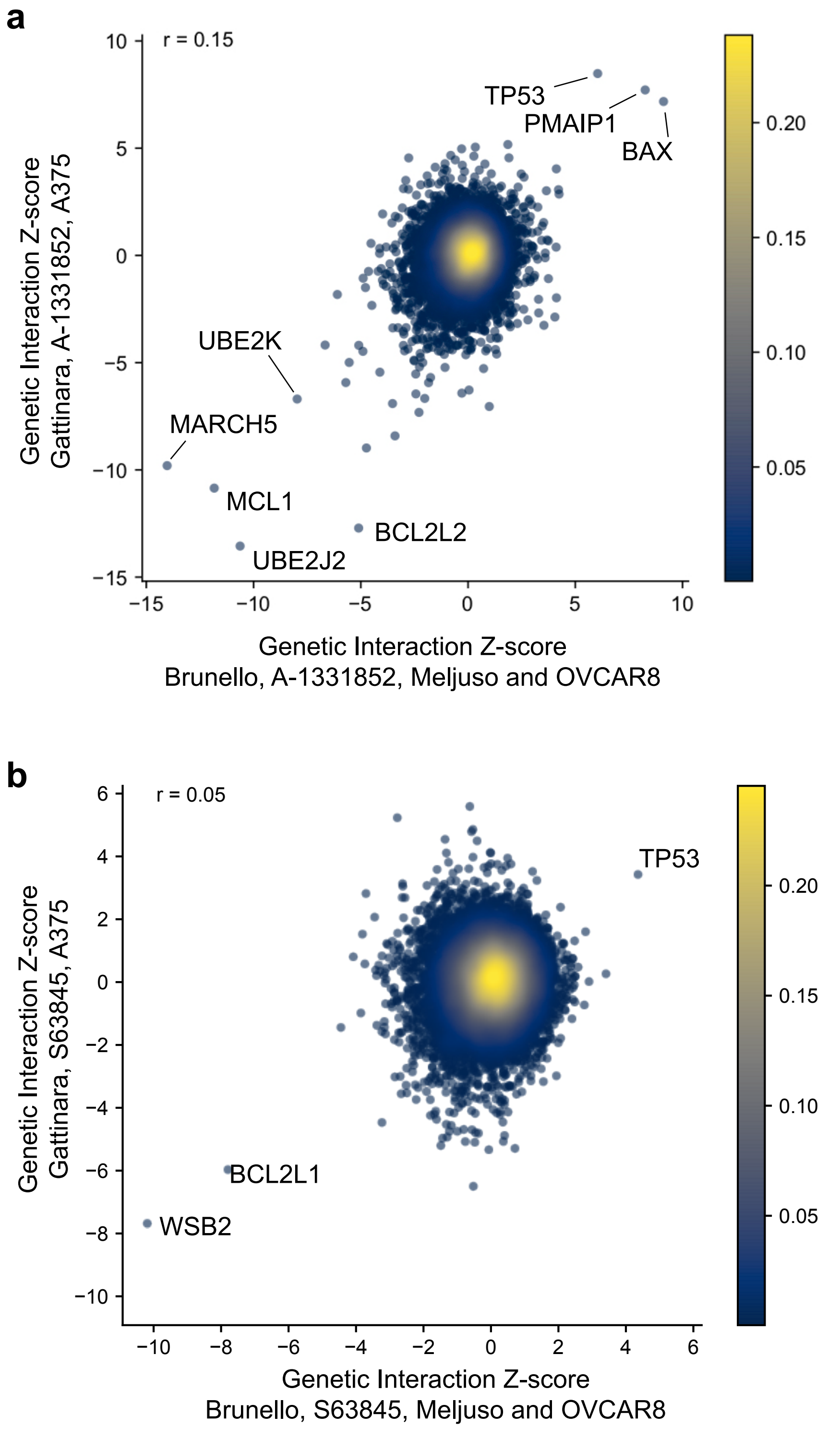

Supplementary Figure 9 Comparison of Gattinara and Brunello screens. (**a**) Z-scores for A-1331852 screened with Brunello and averaged for Meljuso and OVCAR8 cells compared to Z-scores for A-1331852 screened with Gattinara. All genes with an absolute average Z-score greater than 5 are labeled. (**b**) Z-scores for S63845 screened with Brunello and averaged for Meljuso and OVCAR8 cells compared to Z-scores for S63845 screened with Gattinara in A375. Points are colored by density. Pearson correlation coefficient is indicated.

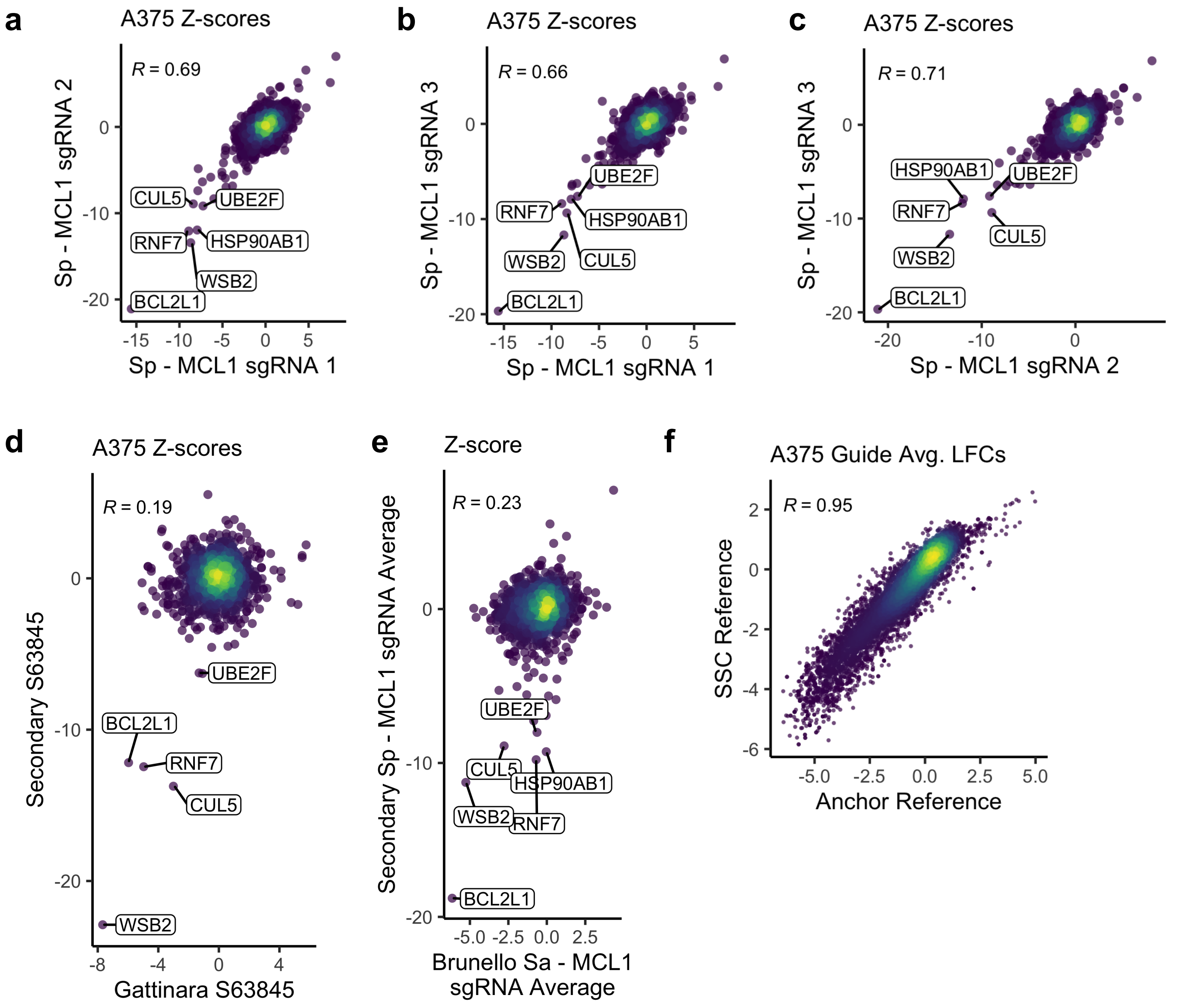

Supplementary Figure 10 Secondary screens are consistent within a perturbation type. (**a**) Comparison of Z-scores for MCL1 anchor guide 2 vs MCL1 anchor guide 1 screened with the secondary library in A375 cells. Pearson correlation is reported and points are colored by density. (**b**) Same as (a) but for guide 3 vs guide 1. (**c**) Same as (a) but for guide 3 vs guide 2. (**d**) Comparison of Z-scores for S63845 screened with Gattinara and the secondary library in A375. Pearson correlation is reported and points are colored by density. (**e**) Comparison of Z-scores for MCL1 Spyo anchors, averaged across all guides and screened with the secondary library in A375 cells vs MCL1 Saur anchor, screened with the Brunello library and averaged across OVCAR8 and Meljuso cell lines. Points are colored by density. Pearson correlation coefficient is indicated. (**f**) Log2-fold changes for the A375 parental line used as a reference for the single cell clones (SSC) compared to the A375 parental line used as a reference for the anchor and S63845 arms, both screened with the secondary library. Points are colored by density. Pearson correlation coefficient is indicated.

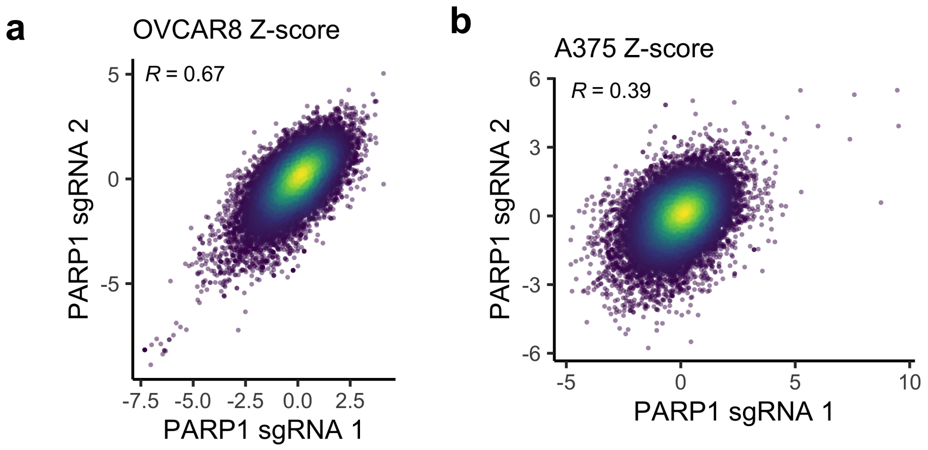

Supplementary Figure 11 PARP1 anchor guide comparison. (**a**) Comparison of Z-scores for PARP1 guide 2 vs PARP1 guide 1 screened with Brunello in OVCAR8. Pearson correlation coefficient is reported and points are colored by density. (**b**) Same as (a) but in A375.

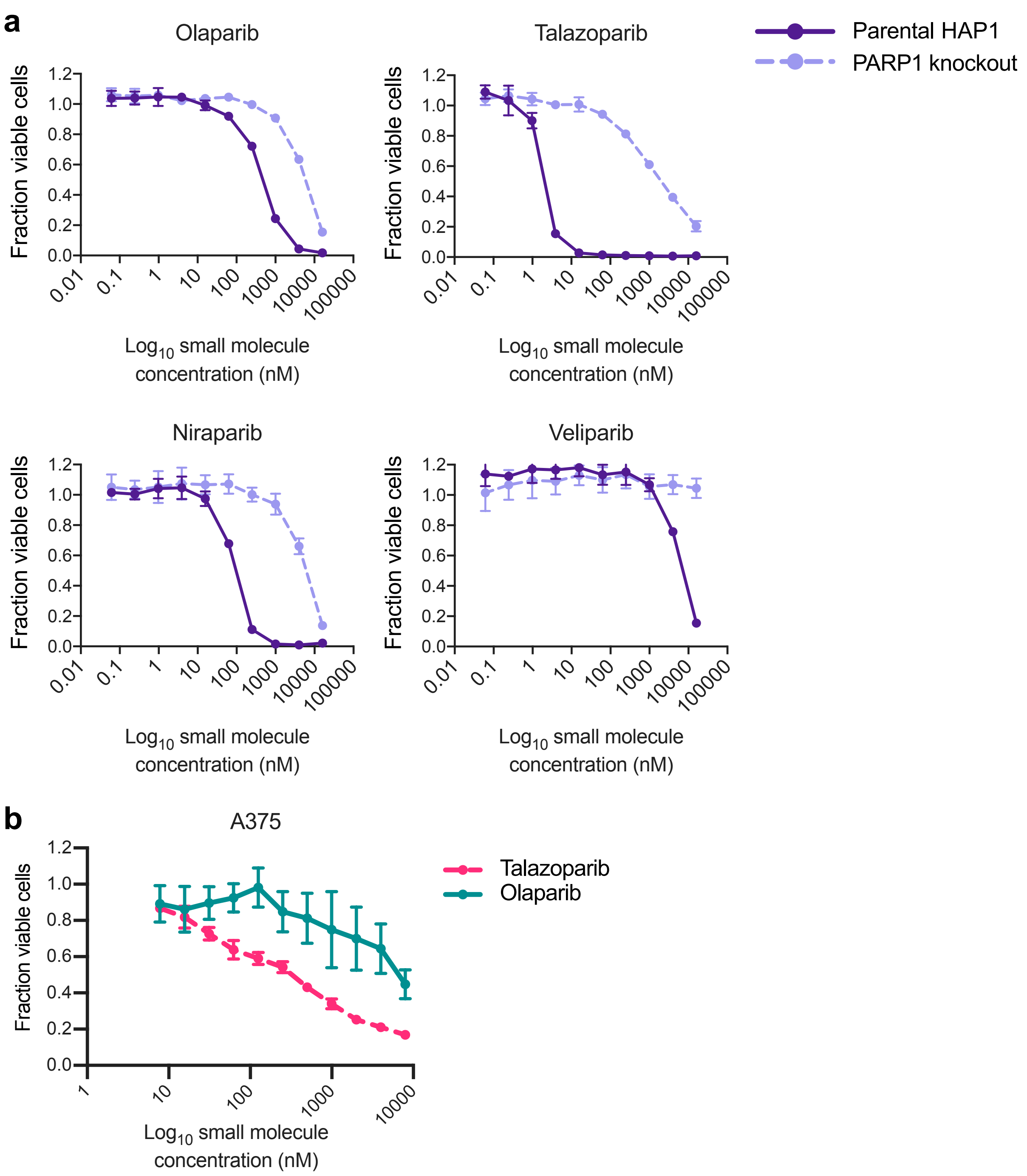

Supplementary Figure 12 Dose response curves with PARP inhibitors. (**a**) Titration of 4 PARP inhibitors in PARP1 knockout single cell clone and parental HAP1 cells. Cells were incubated with respective small molecules for 3 days before assaying viability by Cell Titer Glo. Data are normalized to the zero dose. Points represent the average and whiskers represent the standard deviation of ten replicate wells (n=10). (**b**) Titration of 2 PARP inhibitors in A375 cells. Cells were incubated with respective small molecules for 3 days before assaying viability by Cell Titer Glo. Data are normalized to the zero dose. Points represent the average and whiskers represent the maximum and minimum of duplicate wells.

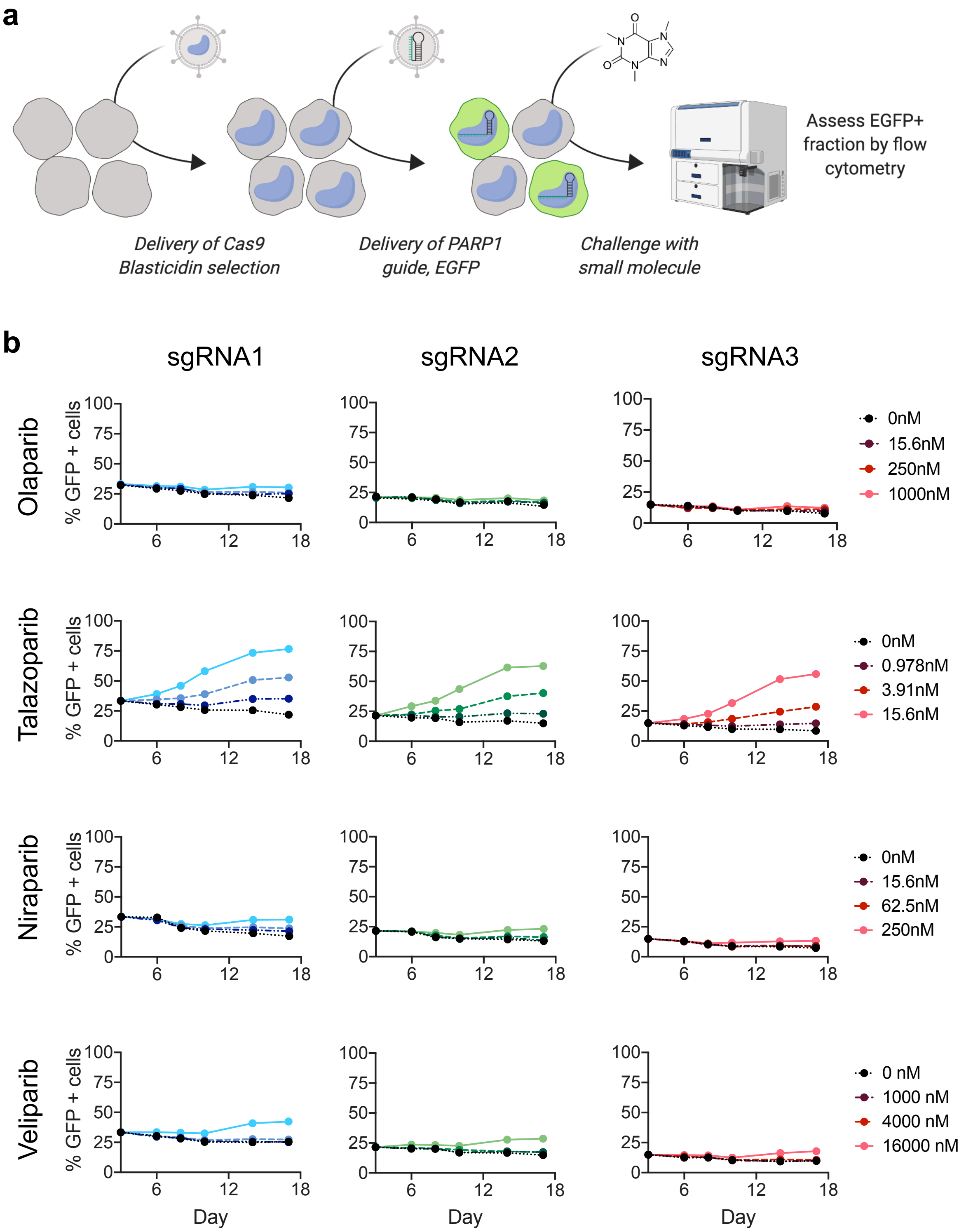

Supplementary Figure 13 Competition assay in A375 cells across PARP inhibitors. (**a**) Competition assay schematic. Cas9 is first introduced to cells, followed by the delivery of 3 unique PARP1 guides in a vector that also confers EGFP expression. Small molecule is then introduced on day 3, and the percentage of EGFP + cells is monitored over time by flow cytometry. (**b**) Percentage of EGFP+ cells over time by guide (column) and small molecule (row). Note that in the screen presented in Figure 6, A375 cells were screened with olaparib at 250 nM and talazoparib at 7.8 nM.

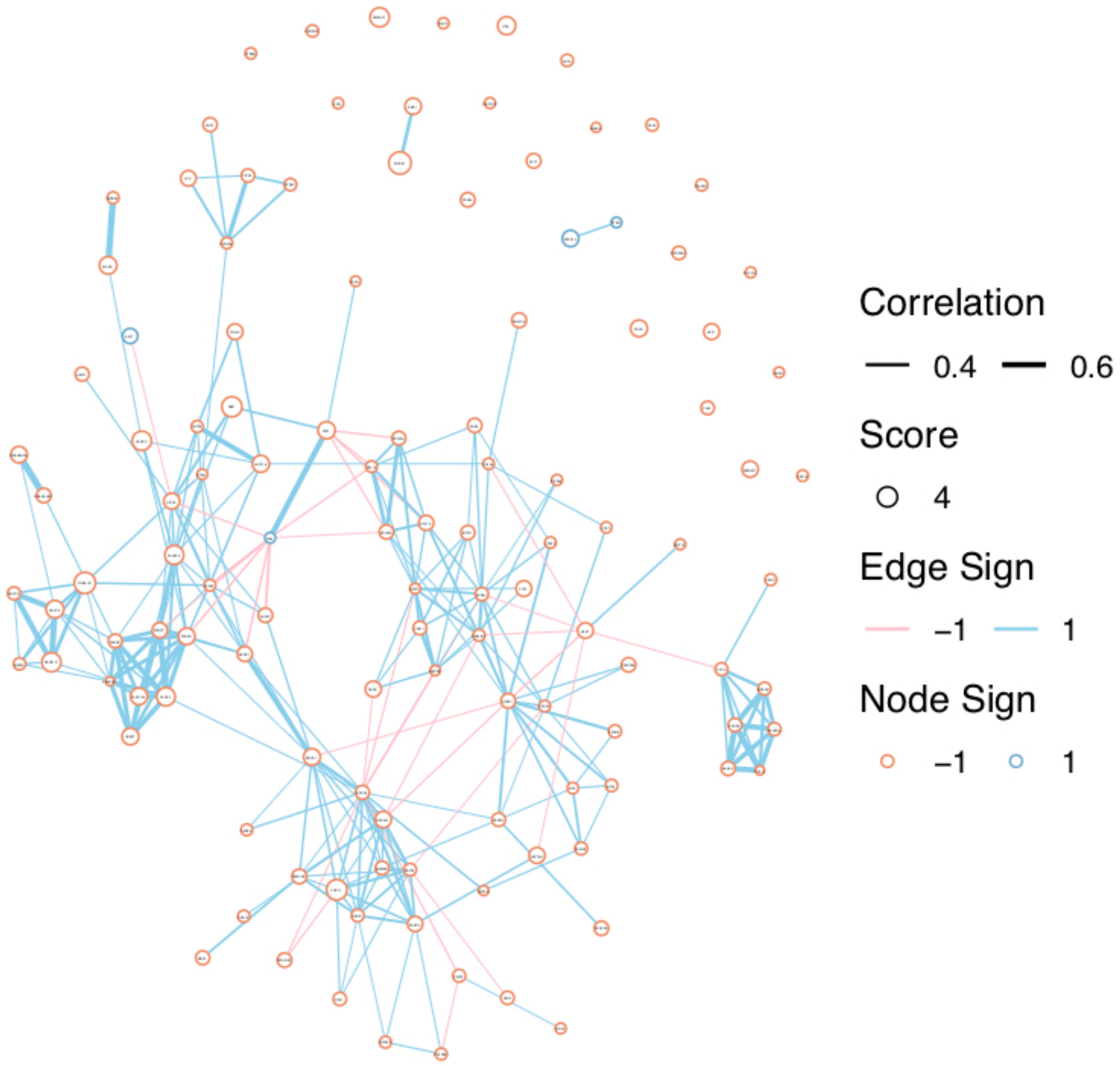

Supplementary Figure 14 Co-essentiality correlations from DepMap connect functionally related gene hits from PARP1 anchor screens. Nodes represent genes and the size of each node is proportional to its average Z-score across all screens. Genes with an absolute average Z-score greater than 2 across PARP1 conditions are included in the network. Edges represent Pearson correlations across co-essentiality profiles in DepMap. Edges are drawn between genes with an absolute correlation greater than 0.2.

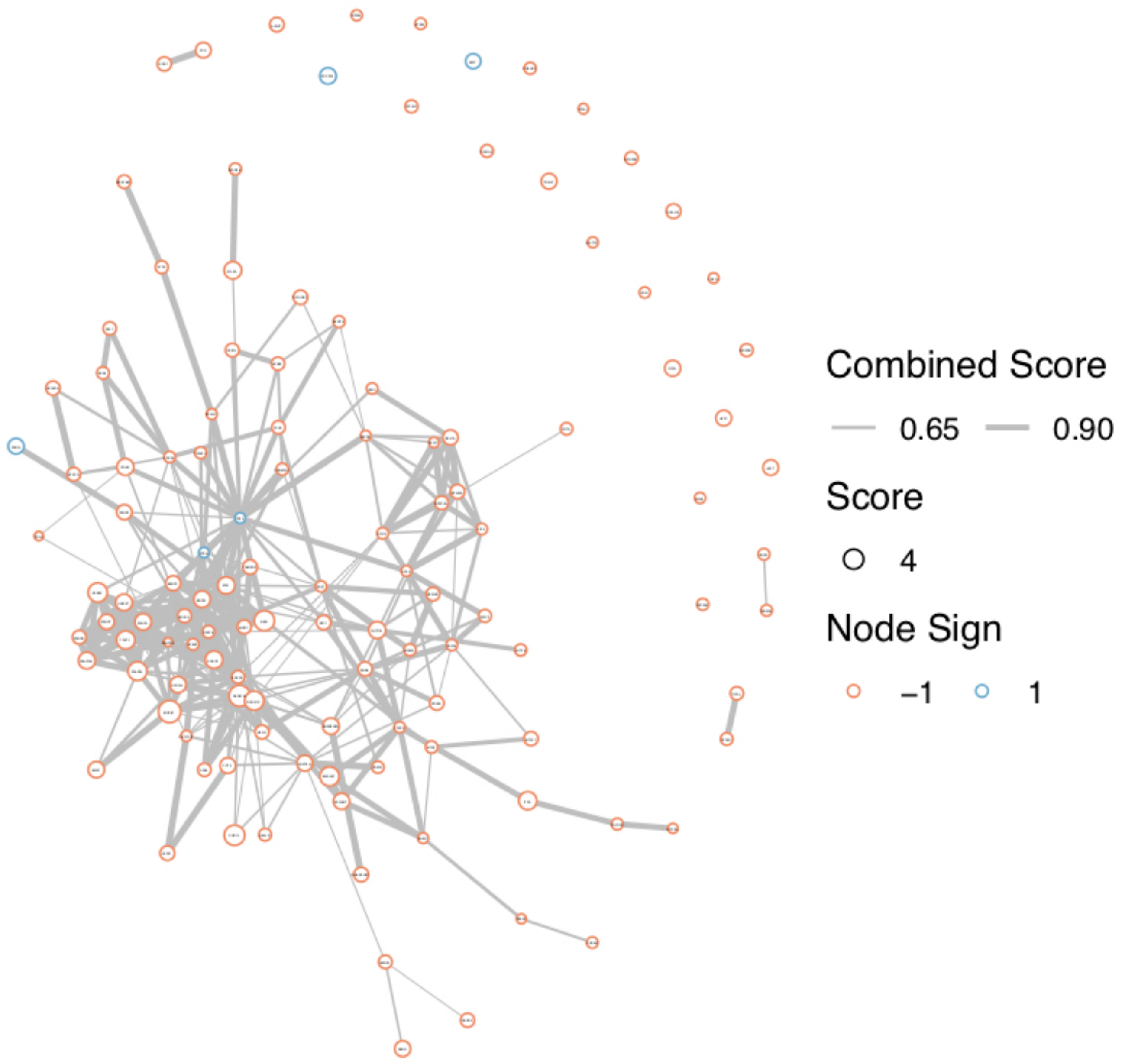

Supplementary Figure 15 Combined scores from STRING connect functionally related gene hits from PARP1 anchor screens. Nodes represent genes and the size of each node is proportional to its average Z-score across all screens. Genes with an absolute average Z-score greater than 2 across PARP1 conditions are included in the network. Edges represent combined score in STRING. Edges are drawn between genes with a STRING combined score greater than 0.4.

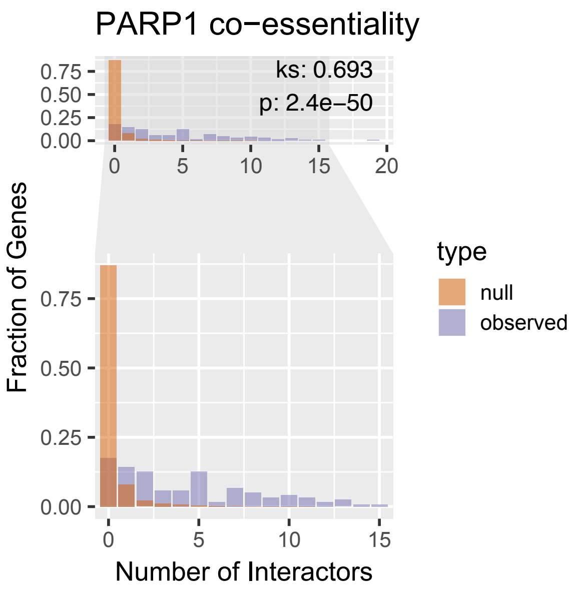

Supplementary Figure 16 Hits from the PARP1 anchor screens are enriched in interactions. Degree distribution for the observed network of PARP1 hits compared with a null distribution using DepMap co-essentialities and STRING combined scores. We average 1,000 random networks, each of which has the same number of genes as the original network (n=122), to generate the null. To determine statistical significance, we used a one-sided KS test with the alternative hypothesis that the observed cumulative distribution was less than the null.

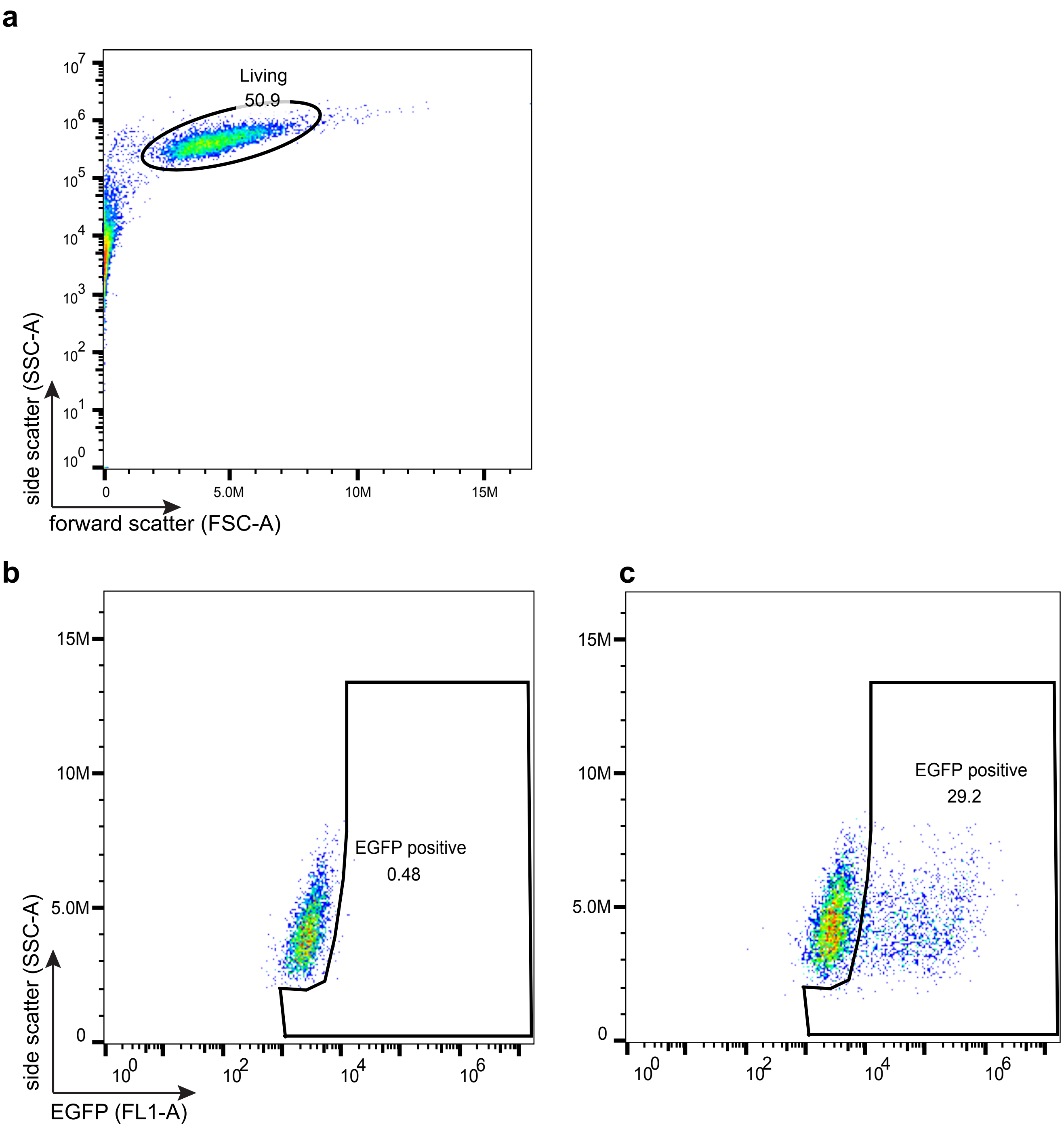

Supplementary Figure 17 Flow cytometry gating strategy for EGFP competition assay. (**a**) The live cell population was first gated using forward scatter and side scatter in parental cells. (**b**) The EGFP-positive population was subsequently gated based on parental (EGFP-negative) cells. (**c**) Representative plot showing the mixed population containing both EGFP-positive and EGFP-negative cells.

Cell Line	Control	Genetic Knockout					Small molecule inhibition			
		MCL1 guide 1	BCL2L1 guide 1	PARP1 guide 1	PARP1 guide 2	Single cell clone	S63845	A133	Olap.	Talaz.
A375	B, G<br>0.84, 0.86			B<br>0.68	B<br>0.85		G<br>0.85	G<br>0.84	B, G<br>0.80, 0.85	G<br>0.79
HAP1	B<br>0.88					B<br>0.92				B<br>0.86
Meljuso	B<br>0.86	B<br>0.79	B<br>0.72				B<br>0.86	B<br>0.79		
OVCAR8	B<br>0.57	B<br>0.72	B<br>0.62	B<br>0.69	B<br>0.74		B<br>0.59	B<br>0.60	B<br>0.70	

Supplementary Table 1. Genome-wide screens in this study. Pearson correlation coefficients are shown for the log2-fold-change relative to the plasmid DNA for replicate screens.

B = Brunello library
G = Gattinara library
A133 = A-1331852
Olap. = Olaparib
Talaz. = Talazoparib

Name	sgRNA Sequence	Figure
MCL1 Saur-guide	CACCCTCACGCCAGACTCCCG	Fig. 2
BCL2L1 Saur-guide	AAGCGCTGAGGGAGGCAGGCG	Fig. 2
MCL1 Spyo-guide 1	AGGAGGAGGACGAGTTGTAC	Fig. 4
MCL1 Spyo-guide 2	GATTATCTCTCGGTACCTTC	Fig. 4
MCL1 Spyo-guide 3	GACTGGCTAGTTAAACAAAG	Fig. 4
PARP1 Saur-guide 1	GGAAGTAAAGGAAGCCAACAT	Fig. 5
PARP1 Saur-guide 2	AGACACAGACACCCAACCGGA	Fig. 5
MCL1 Spyo-guide	AGGCGCTGGAGACCTTACGA	Supp. Fig. 8
BCL2L1 Spyo-guide	CTCCGATTCAGTCCCTTCTG	Supp. Fig. 8
MARCH5 Spyo-guide	CCAGGCCTGTCTACAACGCT	Supp. Fig. 8
WSB2 Spyo-guide	CTTGCTACGGGACTCAACGA	Supp. Fig. 8
PARP1 Spyo-guide 1	CGATGCCTATTACTGCACTG	Supp. Fig. 13
PARP1 Spyo-guide 2	TACCGATCACCGTACCCACA	Supp. Fig. 13
PARP1 Spyo-guide 3	AGCTAGGCATGATTGACCGC	Supp. Fig. 13

Supplementary Table 2 Individual guide sequences used in this study.

Supplementary Note 1

To reduce the cost of executing genome-wide CRISPR screens, we designed a human genome-wide library, Gattinara, with 2 guides per gene. This library was designed to be backwards-compatible with Brunello, which has 4 guides per gene, such that screening with both libraries would total 6 unique guides per gene. This library also targets more protein coding genes than Brunello, due to changes in genome annotation over time.

Gattinara has a total of 40,964 guides targeting 19,993 protein-coding genes. The library also includes 500 non-targeting control guides, as well as 500 guides targeting one intergenic region in the genome to serve as controls for dsDNA breaks. The cumulative distribution function of the plasmid DNA has an AUC of 0.56, indicating a well-made library (**Supplementary Figure 18**).

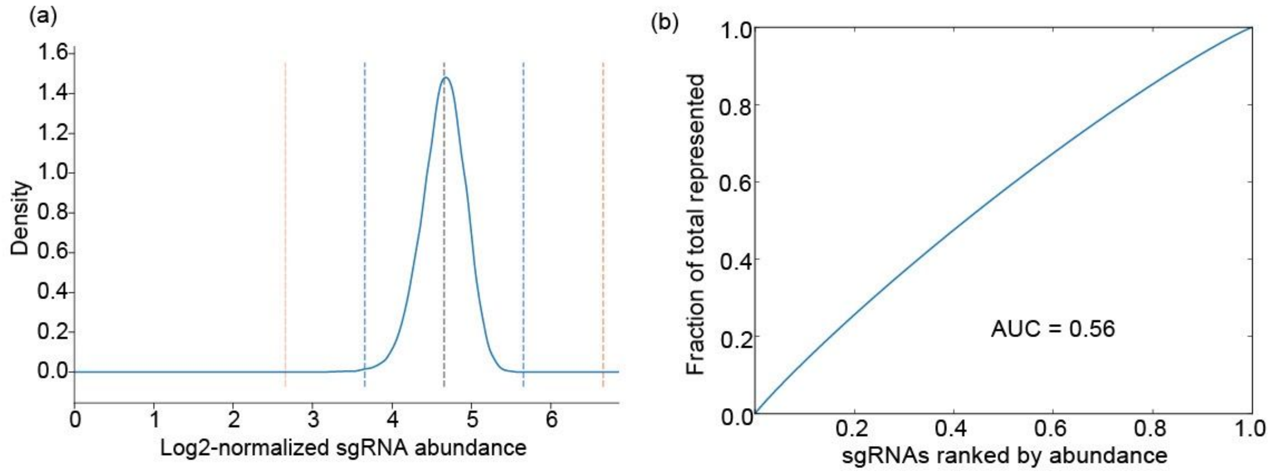

Supplementary Figure 18 Distribution of the Gattinara library. (**a**) Probability distribution function of the log2-normalized values of sgRNA abundance for Gattinara plasmid DNA. The grey dashed line represents the median. The blue dashed lines represent +/- 2-fold of the median and encompass 99.7% of the guides. The orange dashed lines represent +/- 4-fold of the median and encompass 99.9% of the guides. (**b**) Same data as in (**a**) but plotted as a cumulative distribution function.

To compare the performance of Gattinara to other genome-wide libraries, we performed viability screens in A375 cells and compared to existing data in which A375 cells were screened with previous libraries. The dAUC, which measures the separation between guides targeting essential and non-essential genes, is 0.46 for both Gattinara and Brunello, indicating that Gattinara performs equally-well at the guide level (**Supplementary Fig. 19**).

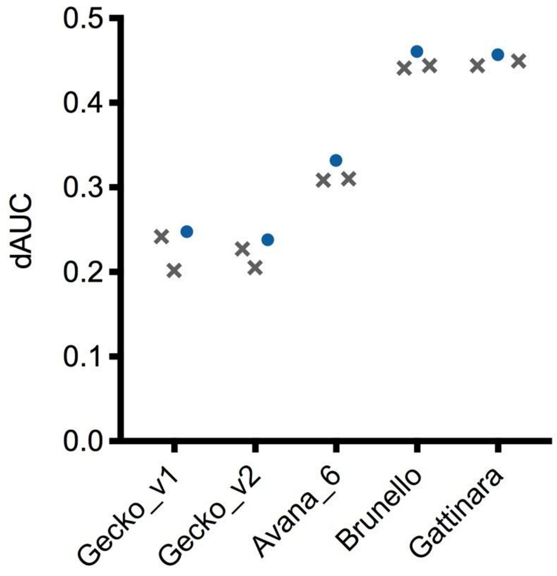

Supplementary Figure 19 Comparison of dAUC across different CRISPRko libraries. The dAUCs of individual replicates are plotted as Xs and dAUCs of combined replicates are plotted as circles.

We then averaged log2-fold changes of guides targeting the same gene and calculated an ROC-AUC, using essential genes as true positives and non-essential genes as false positives. The ROC-AUC for Gattinara is 0.97, compared with 0.98 for Brunello, indicating that we can achieve a gene-level performance similar to Brunello with only 2 sgRNAs per gene. Gattinara outperforms other libraries by both dAUC (guide level) and ROC-AUC (gene level) (**Supplementary Figure 20**).

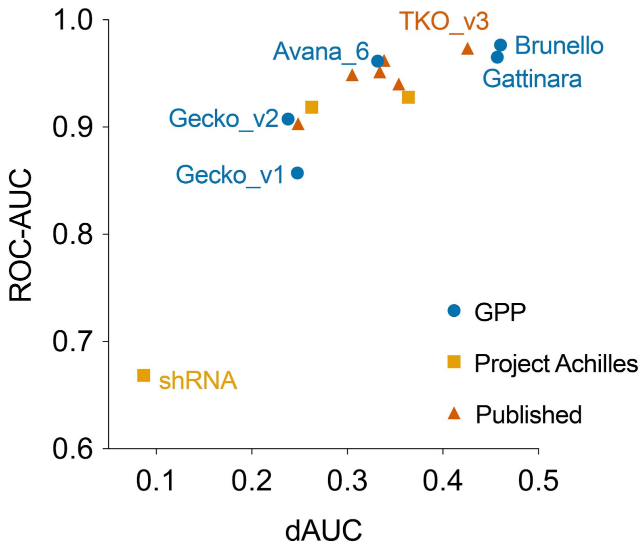

Supplementary Figure 20 Comparison of libraries.

To understand how additional guides affect library performance we randomly sampled a subset of guides for each gene and calculated a gene-level ROC-AUC. We saw that Gattinara with one or two guides performs comparably to Brunello with one or two guides. When we combined the log2-fold

changes from Gattinara and Brunello and performed subsampling analysis, we saw diminishing returns with more than 4 guides per gene (**Supplementary Figure 21**).

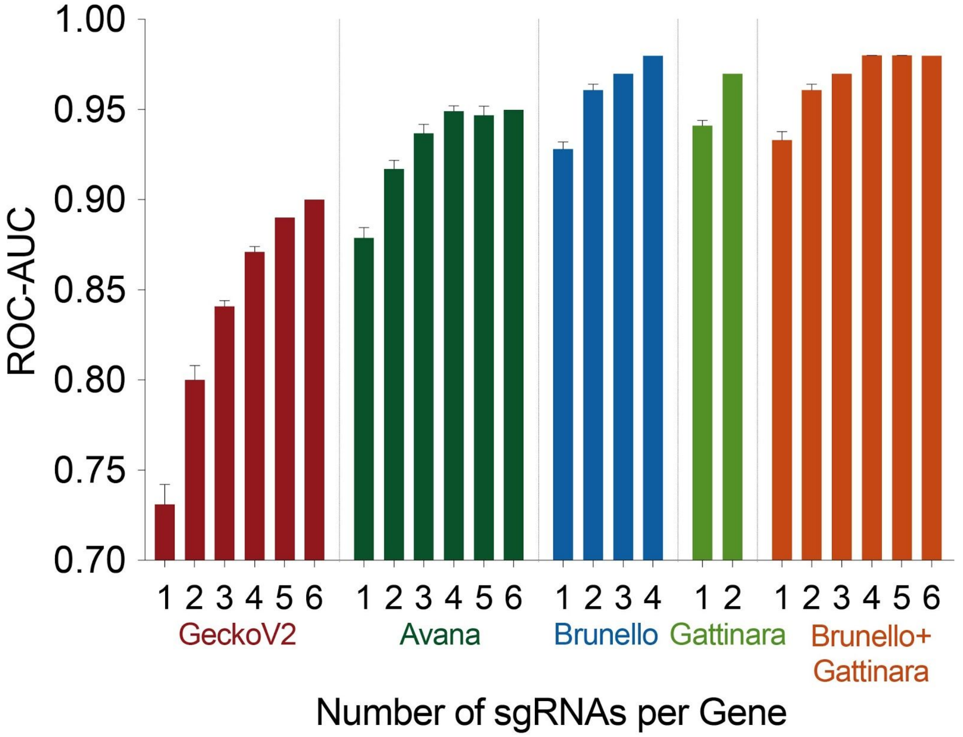

Supplementary Figure 21 ROC-AUCs for subsampled libraries. Bars represent mean values +/- one standard deviation, calculated from n=10 different iterations of resampling without replacement for each library size. For the max number of guides per gene the bar represents the observed ROC-AUC for the library.

Due to gene-specific constraints (small gene size, paucity of NGG PAM sequences), 622 genes in Gattinara had one or two overlapping guides with Brunello. The log2-fold changes of the 865 overlapping guides are well-correlated, with a Pearson coefficient of 0.85 (**Supplementary Figure 22**).

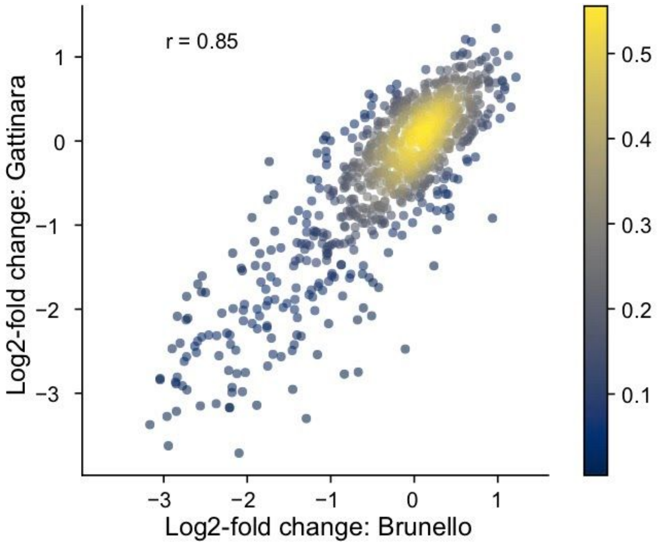

Supplementary Figure 22 Scatter plot showing the log2-fold change of guides that are common between Brunello and Gattinara. Points are colored by density. Pearson correlation coefficient is indicated.

For all genes, the log2-fold changes (average of all guides targeting a gene) showed good correspondence between Gattinara and Brunello, with a Pearson correlation of 0.79 (**Supplementary Figure 23**).

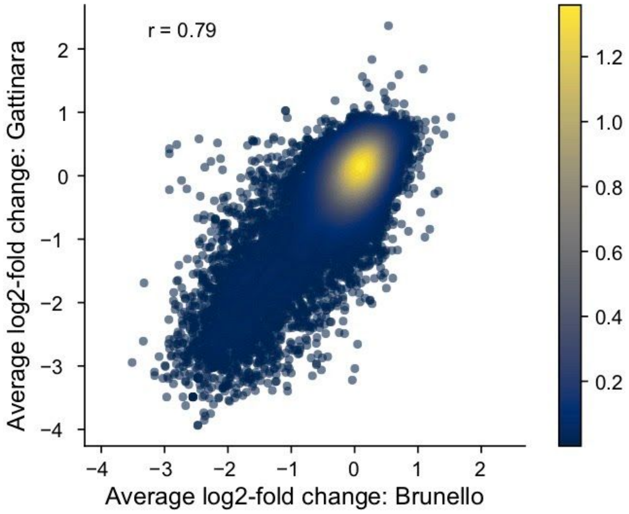

Supplementary Figure 23 Scatter-plot showing the average log2-fold changes of genes in Brunello and Gattinara. Points are colored by density. Pearson correlation coefficient is indicated.

This library is available from Addgene (136986) in a modified version of lentiGuide, pRDA_118 (elimination of the 4 thymidine run in the tracrRNA, plus the addition of convenient restriction sites elsewhere in the vector). We also created a mouse version with the same design criteria, Gouda (Addgene 136987).
