# Supplementary material for: Genetic screens in isogenic mammalian cell lines without single cell cloning
Source: Nat Commun. 2020 Feb 6;11:752. doi: 10.1038/s41467-020-14620-6 (PMC7005275; doi:10.1038/s41467-020-14620-6)
Supplement: Supplementary file 3 — Description of Additional Supplementary Files [file 41467_2020_14620_MOESM3_ESM.pdf]

**Title:** Supplementary Dataset 1
**Description:** Read counts of all screens in this study.

**Title:** Supplementary Dataset 2
**Description:** Gene-level analysis of all screens using the Brunello library.

**Title:** Supplementary Dataset 3
**Description:** Gene-level analysis of all screens using the Gattinara library.

**Title:** Supplementary Dataset 4
**Description:** Gene-level analysis of all screens using the secondary library.

**Title:** Supplementary Dataset 5
**Description:** Collation of gene sets used in this study
