# Supplementary material for: Genetic screens in isogenic mammalian cell lines without single cell cloning
Source: Nat Commun. 2020 Feb 6;11:752. doi: 10.1038/s41467-020-14620-6 (PMC7005275; doi:10.1038/s41467-020-14620-6)
Supplement: Supplementary file 9 — Reporting Summary [file 41467_2020_14620_MOESM9_ESM.pdf]

nature research

Corresponding author(s): Doench

Last updated by author(s): 01/16/2020

Reporting Summary

Nature Research wishes to improve the reproducibility of the work that we publish. This form provides structure for consistency and transparency in reporting. For further information on Nature Research policies, see Authors & Referees and the Editorial Policy Checklist .

Statistics

For all statistical analyses, confirm that the following items are present in the figure legend, table legend, main text, or Methods section.

n/a	Confirmed	
☐	☒	The exact sample size (*n*) for each experimental group/condition, given as a discrete number and unit of measurement
☐	☒	A statement on whether measurements were taken from distinct samples or whether the same sample was measured repeatedly
☐	☒	The statistical test(s) used AND whether they are one- or two-sided<br>*Only common tests should be described solely by name; describe more complex techniques in the Methods section.*
☒	☐	A description of all covariates tested
☐	☒	A description of any assumptions or corrections, such as tests of normality and adjustment for multiple comparisons
☐	☒	A full description of the statistical parameters including central tendency (e.g. means) or other basic estimates (e.g. regression coefficient) AND variation (e.g. standard deviation) or associated estimates of uncertainty (e.g. confidence intervals)
☐	☒	For null hypothesis testing, the test statistic (e.g. *F*, *t*, *r*) with confidence intervals, effect sizes, degrees of freedom and *P* value noted<br>*Give P values as exact values whenever suitable.*
☒	☐	For Bayesian analysis, information on the choice of priors and Markov chain Monte Carlo settings
☒	☐	For hierarchical and complex designs, identification of the appropriate level for tests and full reporting of outcomes
☐	☒	Estimates of effect sizes (e.g. Cohen's *d*, Pearson's *r*), indicating how they were calculated

Our web collection on statistics for biologists contains articles on many of the points above.

Software and code

Policy information about availability of computer code

Data collection	No specialized software or custom code was used for data collection.
Data analysis	Screens were analyzed using custom code written in R version 3.5.1 that is available on GitHub. The link to Github is duplicatively provided both here and within the manuscript, per reporting guidelines:<br>https://github.com/PeterDeWeirdt<br><br>R (version 3.5.1), Python (version 2.8), and PRISM Graphpad (version 8) were used for visualization.<br><br>BD Accuri C6 software was used for preliminary analysis of flow cytometry on the machine itself, but final plots were made in FloJo (version 10), which is necessary to provide the density plots required to comply with the reporting guidelines.

For manuscripts utilizing custom algorithms or software that are central to the research but not yet described in published literature, software must be made available to editors/reviewers. We strongly encourage code deposition in a community repository (e.g. GitHub). See the Nature Research guidelines for submitting code & software for further information.

Data

Policy information about availability of data

All manuscripts must include a data availability statement. This statement should provide the following information, where applicable:

- Accession codes, unique identifiers, or web links for publicly available datasets
- A list of figures that have associated raw data
- A description of any restrictions on data availability

Here we provide a duplicate of the data availability statement from the manuscript:
The read counts for all screening data and subsequent analyses are provided as Supplementary Data. Fastq files of sequencing are available from the Sequencing Read Archive, accession code SRP217813.

Field-specific reporting

Please select the one below that is the best fit for your research. If you are not sure, read the appropriate sections before making your selection.

☒ Life sciences ☐ Behavioural & social sciences ☐ Ecological, evolutionary & environmental sciences

For a reference copy of the document with all sections, see nature.com/documents/nr-reporting-summary-flat.pdf

Life sciences study design

All studies must disclose on these points even when the disclosure is negative.

Sample size	All screens were performed in duplicate or triplicate. The good correlation between such screens suggests these are adequate sample sizes.
Data exclusions	No data were excluded.
Replication	All screens were performed in at least duplicate, with good correspondence between replicates.
Randomization	Samples were not randomized, because this question does not pertain to any of the science conducted in this manuscript.
Blinding	Samples were not blinded because there was no group allocation for any studies involved here.

Reporting for specific materials, systems and methods

We require information from authors about some types of materials, experimental systems and methods used in many studies. Here, indicate whether each material, system or method listed is relevant to your study. If you are not sure if a list item applies to your research, read the appropriate section before selecting a response.

Materials & experimental systems

n/a	Involved in the study	
☒	☐	Antibodies
☐	☒	Eukaryotic cell lines
☒	☐	Palaeontology
☒	☐	Animals and other organisms
☒	☐	Human research participants
☒	☐	Clinical data

Methods

n/a	Involved in the study	
☒	☐	ChIP-seq
☐	☒	Flow cytometry
☒	☐	MRI-based neuroimaging

Eukaryotic cell lines

Policy information about cell lines

Cell line source(s)	A375, Meljuso, and OVCAR8 - Cancer Cell Line Encyclopedia.<br>HEK293T - ATCC (CRL-3216).<br>HAP1 parental and PARP1-knockout cells; MCL1-knockout clones - Horizon Discovery.
Authentication	A375, Meljuso, OVCAR8, and HEK293T cell lines were authenticated by SNP profiling.<br>HAP1 cells , HAP1-PARP1-ko, and A375-MCL1-ko were generated by Horizon Discovery. They did not provide cell-line-identify authentication information.
Mycoplasma contamination	Cell lines were routinely tested for mycoplasma (~bimonthly). None were mycoplasma positive.
Commonly misidentified lines<br>(See ICLAC register)	None

Flow Cytometry

Plots

Confirm that:

- [x] The axis labels state the marker and fluorochrome used (e.g. CD4-FITC).
- [x] The axis scales are clearly visible. Include numbers along axes only for bottom left plot of group (a 'group' is an analysis of identical markers).
- [x] All plots are contour plots with outliers or pseudocolor plots.
- [x] A numerical value for number of cells or percentage (with statistics) is provided.

Methodology

Sample preparation	Cells were collected from flasks with trypsin and resuspended in flow buffer (PBS supplemented with 2% FBS and 5 µM EDTA).
Instrument	The BD Accuri C6 Sampler was used for all flow cytometry.
Software	The BD Accuri C6 Plus software was used to analyze all flow cytometry data. This software does not support contour plots, so we imported the FCS files into FLoJo (version 10) for the creation of figures.
Cell population abundance	Cells were analyzed only and were not sorted / collected.
Gating strategy	The live cell population was gated using forward- and side-scatter. The gate for EGFP+ cells was set based on parental (EGFP-) cells, and an example is provided as a supplementary figure.

- [x] Tick this box to confirm that a figure exemplifying the gating strategy is provided in the Supplementary Information.
